# Supplementary material for: A quantitative model reveals a frequency ordering of prediction and prediction-error signals in the human brain
Source: Commun Biol. 2022 Oct 10;5:1076. doi: 10.1038/s42003-022-04049-6 (PMC9550773; doi:10.1038/s42003-022-04049-6)

Supplementary Information:

A quantitative model reveals a frequency ordering of prediction and prediction-error signals in the human brain

Zenas C. Chao[1]*, Yiyuan Teresa Huang[1,2], Chien-Te Wu[1,2]

1. International Research Center for Neurointelligence (WPI-IRCN), UTIAS, The University of Tokyo, Tokyo, Japan
2. School of Occupational Therapy, College of Medicine, National Taiwan University, Taipei, Taiwan

***Correspondance:**

Zenas C. Chao (zenas.c.chao@gmail.com)

Supplementary Figure 1. Overall contrast responses from the within-block and across-block contrasts. (a) The overall occurrence of significant contrast responses for the within-block contrasts (xy – xx and xo – xx). The pixel value represents the ratio of significance across all channels and contrasts. To compare contrast responses in contrasts xy – xx and xo – xx, we evaluated the overall occurrence of contrast responses by averaging the significance (0 or 1) across all channels and all contrasts within contrasts xy – xx and xo – xx separately. The results indicated that contrast responses were stronger in xy – xx than in xo – xx, which was consistent with the model predictions: contrasts 1 to 8 (xy – xx) show bigger sizes than contrasts 9 to 16 (xo – xx) (see Figure 3b). Moreover, contrast responses occurred primarily after the last tone and included both low-frequency (< 10Hz) and high-frequency (> 40Hz) components. (b) The overall occurrence of significant contrast responses for the across-block contrasts, where prominent beta-band responses (13~30Hz) were found.

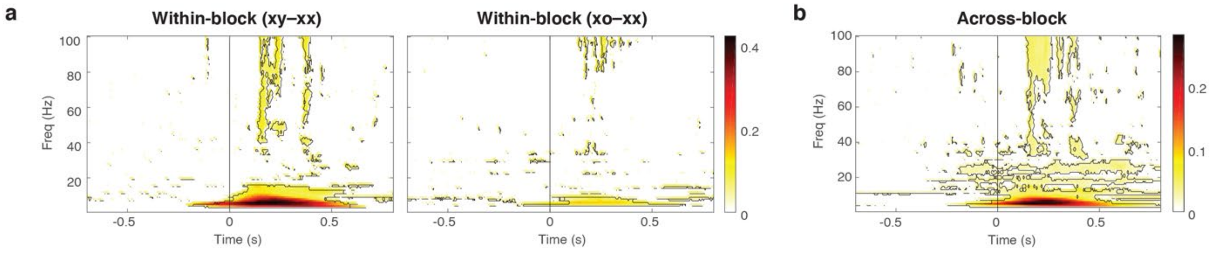

Supplementary Figure 2. Two components extracted from the within-block contrasts by a data-driven analysis. Two components (C1 and C2) were obtained from PRAFAC with 100% consistency, and are visualized by their composition in the three tensor dimensions: *Channel*, *Time-Frequency*, and *Contrast*. The same representation is used as in Figure 5. Note that in the *Contrast* dimension, the data-driven results (blue line) showed patterns similar to the model predictions (orange line), suggesting that C1 and C2 were related to PE1 and PE2, respectively. This also indicated that the proposed model explained the data well (see model comparisons in Figure 4).

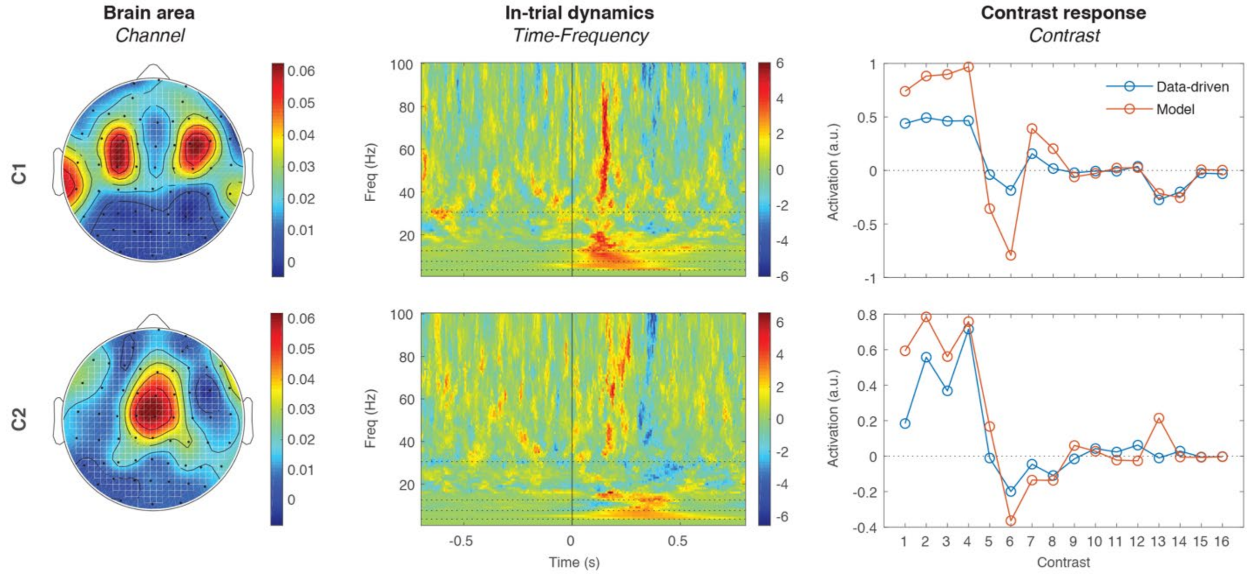

Supplementary Figure 3. The error component in the across-block data-fitting. For data-fitting in the across-block contrasts, we tuned the third component by *a**PE1+(1–*a*)*PE2, where *a* was a weighting factor between 0 and 1. The RSS and the model-fitting consistency are shown. The minimal RSS with a consistency of 85% was found when *a*= 0.5 (equally-weighted PE1 and PE2, as PE1+PE2 in Figure 6).

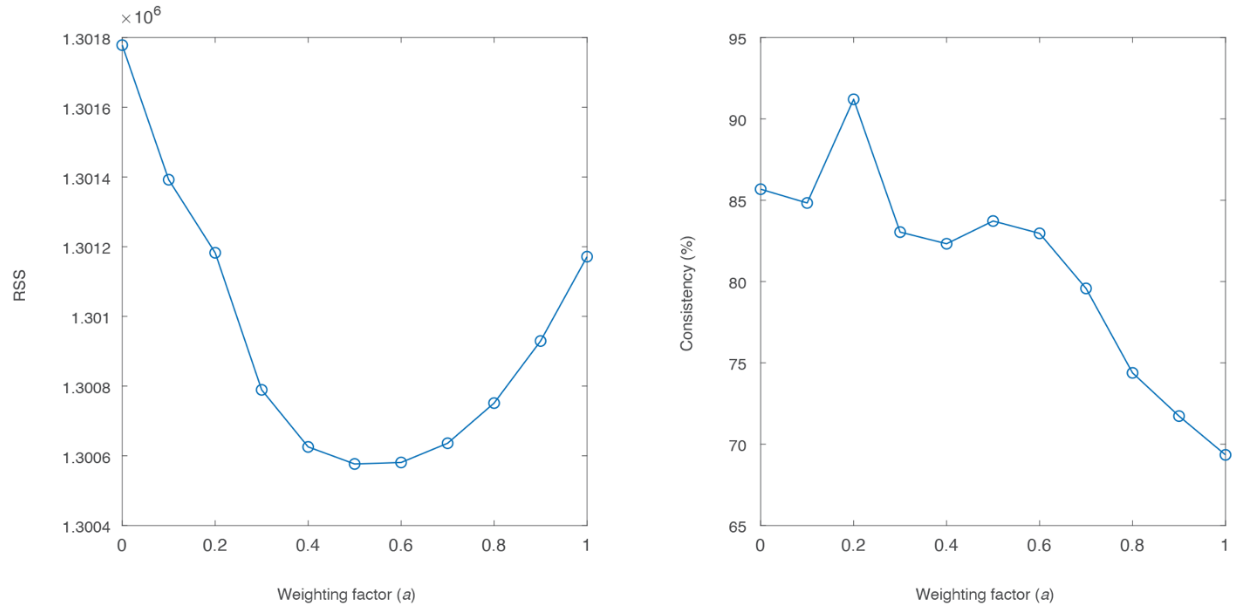

Supplementary Figure 4. Positive and negative prediction errors. The model-driven analysis was performed by using models with different scaling factors s_0 (between 0 and 1), s_1 (between 0 and 2), s_2 (between 0 and 2), and different error types: both positive- and negative-error computations (POS+NEG), only positive-error computation (POS), and only negative-error computation (NEG). (a) The optimal parameters for data-fitting for the within-block contrasts. Only models with a fitting consistency >80% were considered. For each s_0, the minimal residual sum of squares (RSS) across different combinations of s_1 and s_2 is shown in the left panel for POS+NEG (black line), POS (red line), and NEG (blue line). The minimal RSS was found in POS+NEG when s_0= 0.3 (indicated by a black circle). The combination of s_1 and s_2 under this minimum is indicated by a white circle in the right panel. Models with a fitting consistency >80% are indicated by white dots. The color bar represents RSS. The right panel is identical to the one in Figure 4d. (b) The optimal parameters for data-fitting for the across-block contrasts. The same representation is used as in panel a. For NEG, all models showed fitting consistencies below 80%, thus no NEG plotted in the left panel.

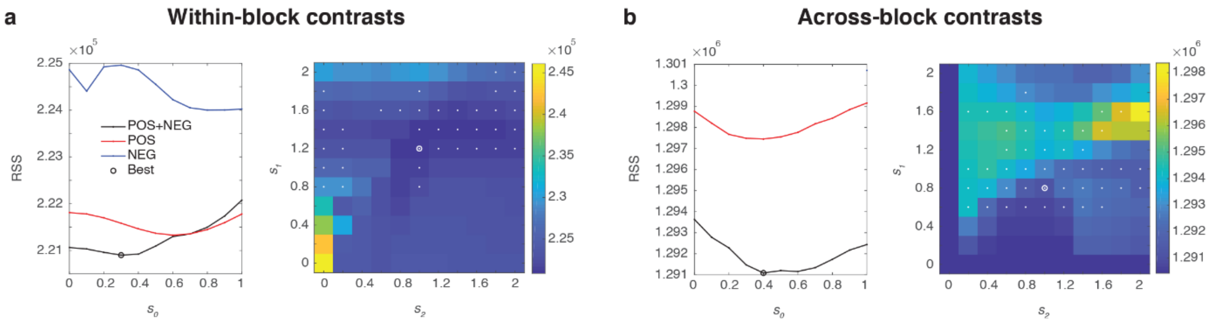

Supplementary Figure 5. Alternative transition probability calculations. (a) The optimal parameters for data-fitting for the within-block contrasts. Only models with a fitting consistency >80% were considered. For each s_0, the minimal RSS across different combinations of s_1 and s_2 is shown in the left panel for the proposed model (black line), *No-ending* (red line), and *No-transition* (blue line). The minimal RSS was found in the proposed model when s_0= 0.3 (indicated by a black circle). The combination of s_1 and s_2 under this minimum is indicated by a white circle in the right panel, which is identical to the one in Figure 4d. (b) The optimal parameters for data-fitting for the across-block contrasts. The same representation is used as in panel a. For *No-ending* (red line), models showed fitting consistencies below 80% when s_0= 1, thus not plotted in the left panel.

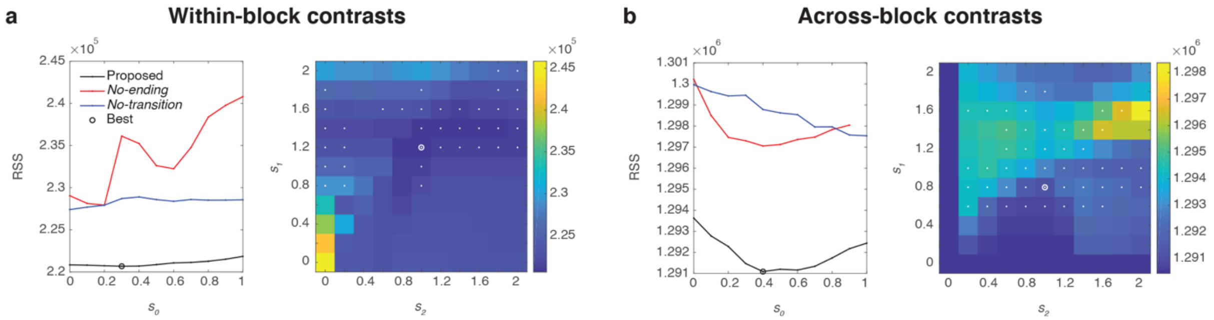

Supplementary Figure 6. Adaptation in auditory responses. (a) Event-related potential (ERP) responses to multi-tone stimulation and adaption factor fitting. ERPs averaged from 180 trials in a single subject are shown in orange ("Actual"). The convolutions of the single-tone ERP response (not shown) with different adaption factor are shown in blue ("Conv"). As shown in the right column where s_0= 1 was used for convolution (no adaptation), the repeats of single-tone responses failed to capture the actual responses. (b) The mean-squared error (MSE) between the actual and convoluted responses under different adaptation factors. For 2-, 3-, 4, and 5-tone sequences, the minimal MSEs were found when s_0= 0.2~0.3.

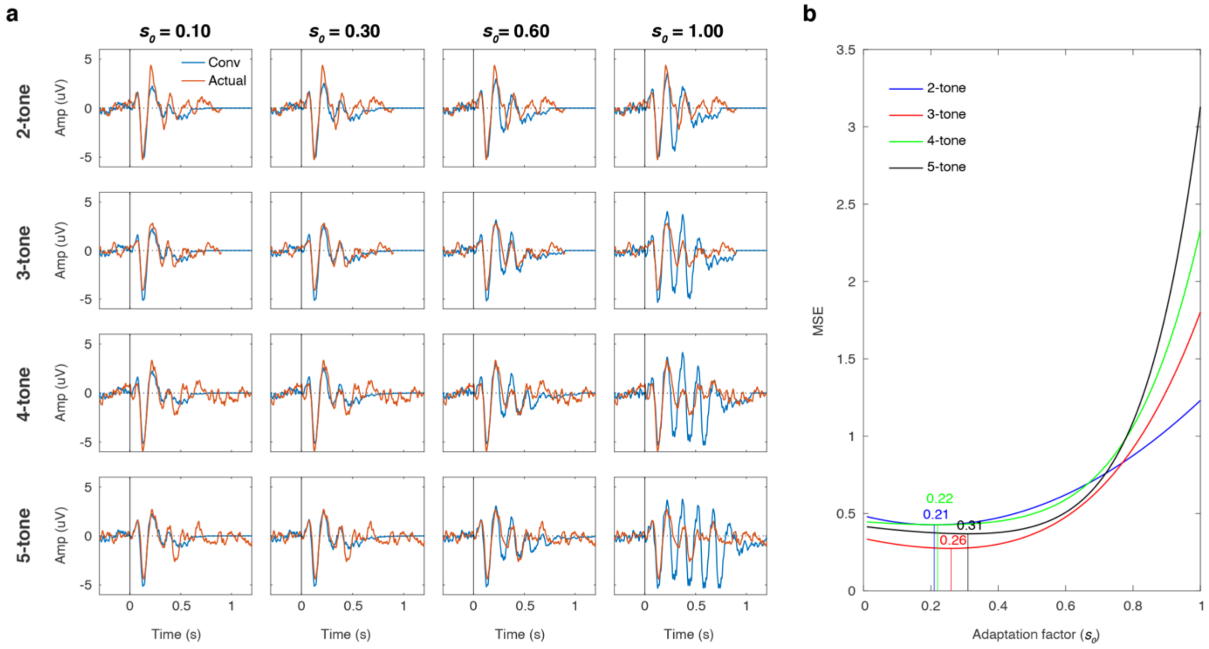

Supplementary Figure 7. Model comparison: one-level predictive-coding models. The model-driven analysis was performed for a single-level predictive-coding model with only transition probability (*1-level PC:TP*), and a single-level predictive-coding model with only sequence probability (*1-level PC:SP*) for the within-block and across-block contrasts. For both models, different scaling factors s_0 (between 0 and 1) and s_1 (between 0 and 2) were evaluated. The combination of s_0 and s_1 with the minimal RSS indicated by a white circle. Models with a fitting consistency >80% are indicated by white dots. The color bar represents RSS.

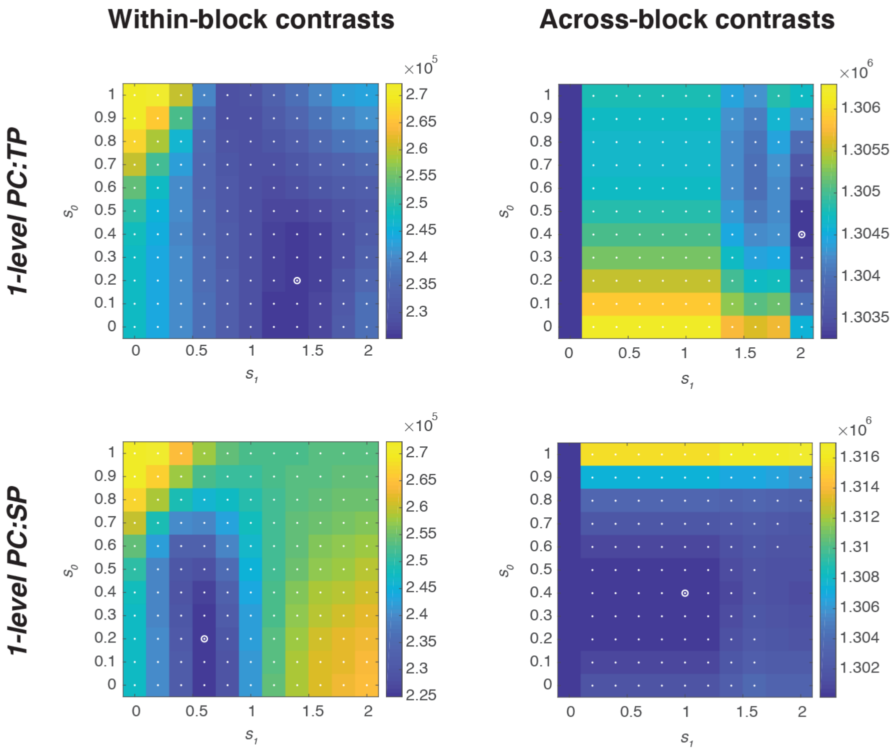

Supplementary Figure 8. Model comparison: adaptation-only model. For the adaptation-only model with no predictive-coding mechanisms (*Adaptation-only*), sensory adaptation was modeled by two parameters: a scaling factor s_0 which determines the maximal capacity for a stimulus to evoke another response immediately after a stimulus, and a time constant τ_0 which determines how fast this capacity recovers. (a) An example of sensory adaption over 144 trials in Block 1 with $s_0 = 0.2$ and $\tau_0 = 1.6$ second. The response capacity adjustment (ΔC, y-axis) over time (x-axis) in the x stream (blue line) and y stream (red line) are shown. After each *x* tone, adaptation occurs in the x stream, where ΔC decreases by s_0, and recovers afterward exponentially with a time constant τ_0. Similarly, adaptation occurs in the y stream after each *y* tone. For each block, we simulated 20 different random sequences, and only one example is shown here. (b) The response to the last tone for each trial (144 trials in total) in a block (the same example as in A). For each trial, the response is determined by ΔC at the time of the last tone ($1 + \Delta C$). That is, if $\Delta C = 0$, then the last tone can evoke a full response. On the other hand, if $\Delta C = -1$, then the last tone cannot evoke any response. Note that for the xo sequence (black line), no response can be evoked. (c) Data-fitting results for the within-block and across-block contrasts across combinations of s_0, and τ_0. The best-fitting model is indicated by a white circle. Models with a fitting consistency >80% are indicated by white dots. The color bar represents RSS.

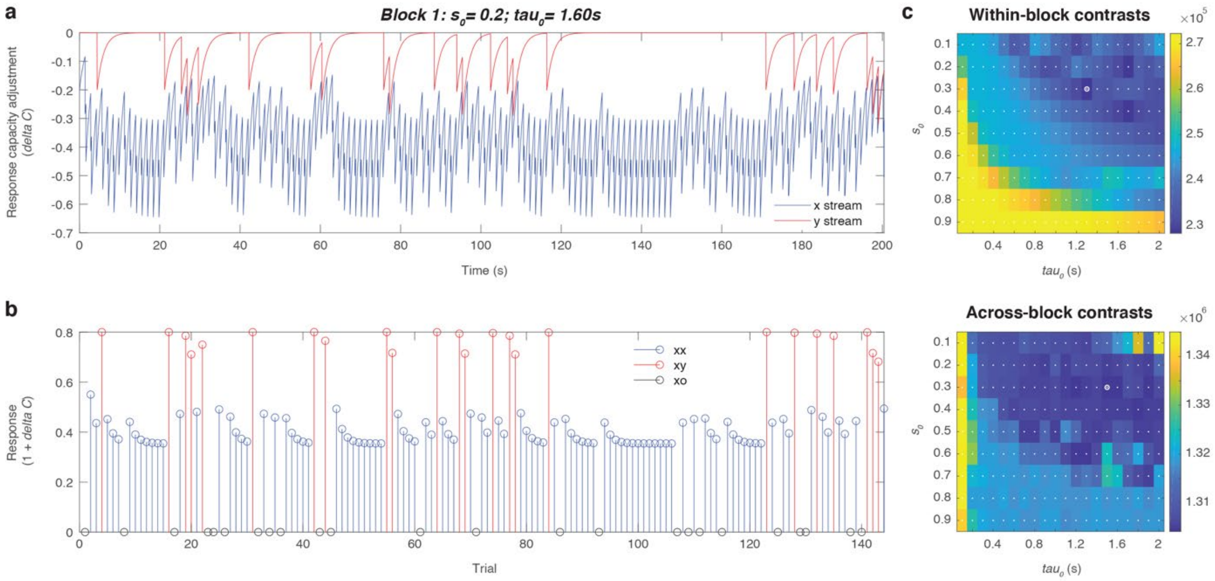

Supplementary Figure 9. The temporal dynamics of PE1 and PE2 in different frequency bands (within-block contrasts). The *Time-Frequency* dimension (Figure 5b) was averaged across frequency bins in different frequency bands, and the temporal dynamics for PE1 (red line) and PE2 (orange line) are shown. The same representation is used as in Figure 5E. The time zero represents the onset of the last tone.

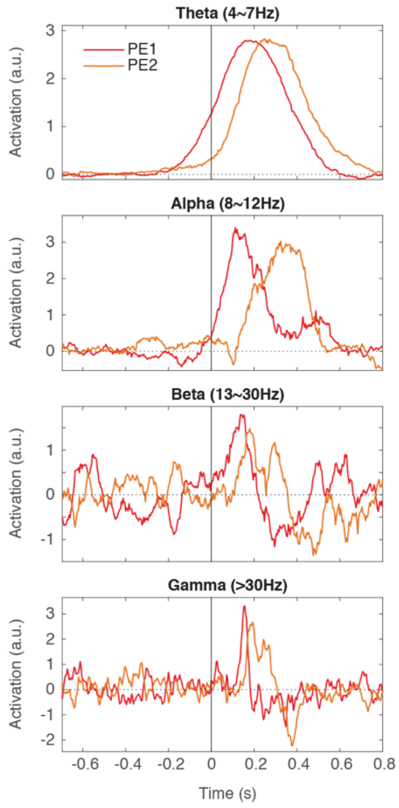

Supplementary Figure 10. Correlation between PE1 and PE2 in within-block and across-block contrasts. The correlation between the (PE1+PE2) component in the across-block contrasts (as in Figure 6) and the sum of PE1 and PE2 components in the within-block contrasts (as in Figure 5). The correlations in the first dimension (*Channel*) and the second dimension (*Time-Frequency*) are shown. In the first dimension, each blue circle represents a channel, and in the second dimension, each blue circle represents a pixel in the Time-Frequency plot.

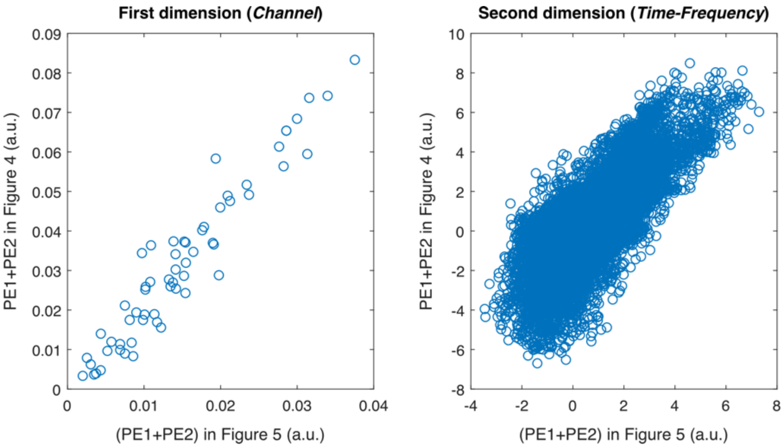

Supplementary Figure 11. Single-trial projection and adjusted correlation. (a) The projection values of PE1, PE2, P1, and P2 over 144 trials in Subject 1, Block 3, and Run 1. (b) The projection values of the same block as in panel a after shuffling. (c) The correlation coefficients *r* between PE1, PE2, P1, and P2 obtained from the projection values in panel a. (d) The correlation coefficients (*r*) between PE1, PE2, P1, and P2 obtained from the projection values in panel b. (e) The difference between panels c and d. The three direct connections shown in Figure 8c are indicated by red circles.

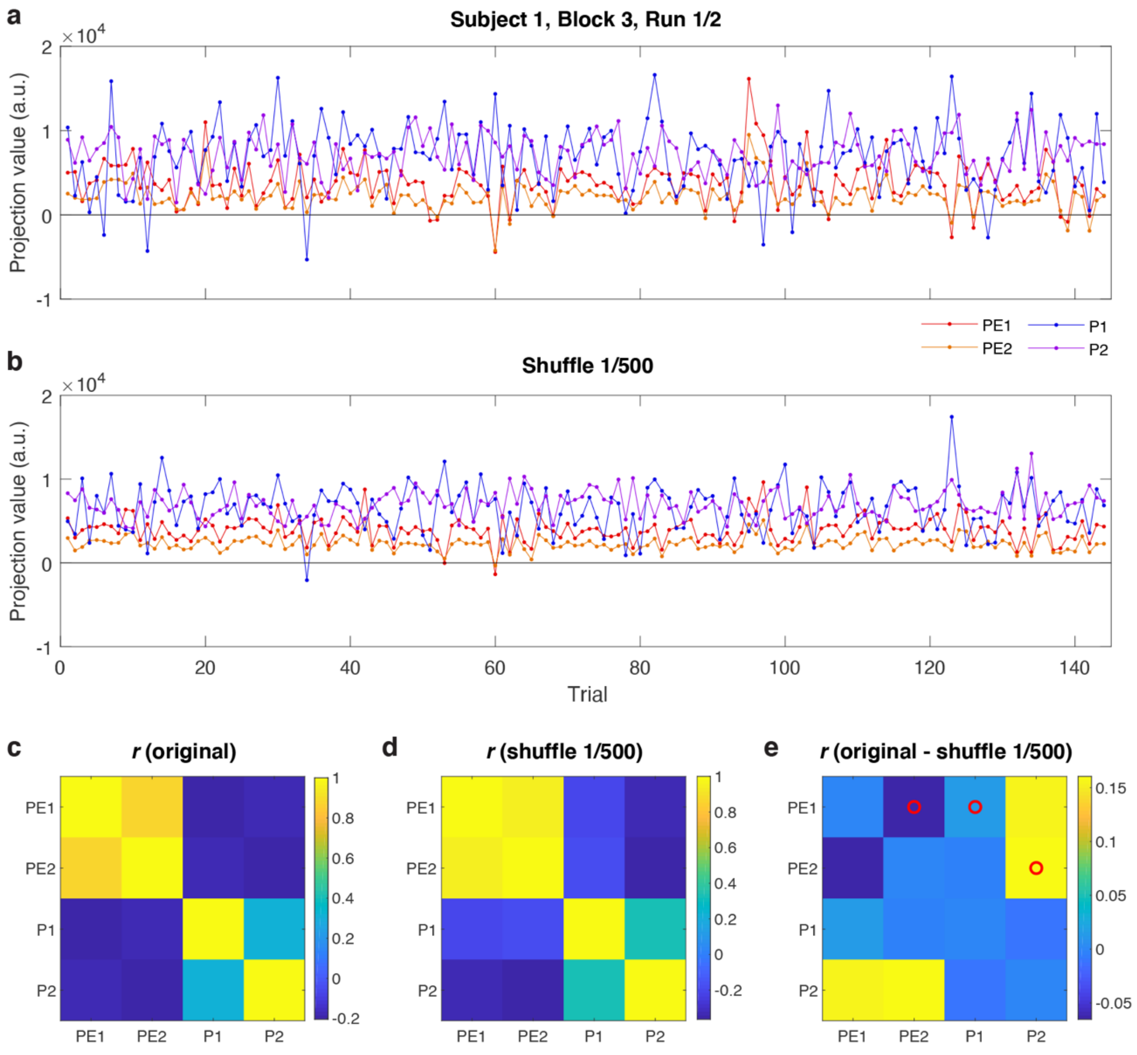

Supplement: Supplementary file 2 — Supplementary Information [file 42003_2022_4049_MOESM2_ESM.pdf]
