# Supplementary material for: A quantitative model reveals a frequency ordering of prediction and prediction-error signals in the human brain
Source: Commun Biol. 2022 Oct 10;5:1076. doi: 10.1038/s42003-022-04049-6 (PMC9550773; doi:10.1038/s42003-022-04049-6)
Supplement: Supplementary file 3 — Description of Additional Supplementary Files [file 42003_2022_4049_MOESM3_ESM.docx]

**Description of Additional Supplementary Files**

**File name:** Supplementary Data 1
**Description:** The source data behind the graphs in the paper.
