# Supplementary material for: A quantitative model reveals a frequency ordering of prediction and prediction-error signals in the human brain
Source: Commun Biol. 2022 Oct 10;5:1076. doi: 10.1038/s42003-022-04049-6 (PMC9550773; doi:10.1038/s42003-022-04049-6)
Supplement: Supplementary file 5 — Reporting summary [file 42003_2022_4049_MOESM5_ESM.pdf]

Corresponding author(s): Zenas C. Chao

Last updated by author(s): Sep 16, 2022

Reporting Summary

Nature Portfolio wishes to improve the reproducibility of the work that we publish. This form provides structure for consistency and transparency in reporting. For further information on Nature Portfolio policies, see our Editorial Policies and the Editorial Policy Checklist.

Statistics

For all statistical analyses, confirm that the following items are present in the figure legend, table legend, main text, or Methods section.

n/a	Confirmed	
☐	☒	The exact sample size (*n*) for each experimental group/condition, given as a discrete number and unit of measurement
☒	☐	A statement on whether measurements were taken from distinct samples or whether the same sample was measured repeatedly
☐	☒	The statistical test(s) used AND whether they are one- or two-sided<br>*Only common tests should be described solely by name; describe more complex techniques in the Methods section.*
☒	☐	A description of all covariates tested
☐	☒	A description of any assumptions or corrections, such as tests of normality and adjustment for multiple comparisons
☐	☒	A full description of the statistical parameters including central tendency (e.g. means) or other basic estimates (e.g. regression coefficient) AND variation (e.g. standard deviation) or associated estimates of uncertainty (e.g. confidence intervals)
☐	☒	For null hypothesis testing, the test statistic (e.g. *F*, *t*, *r*) with confidence intervals, effect sizes, degrees of freedom and *P* value noted<br>*Give P values as exact values whenever suitable.*
☒	☐	For Bayesian analysis, information on the choice of priors and Markov chain Monte Carlo settings
☒	☐	For hierarchical and complex designs, identification of the appropriate level for tests and full reporting of outcomes
☒	☐	Estimates of effect sizes (e.g. Cohen's *d*, Pearson's *r*), indicating how they were calculated

Our web collection on statistics for biologists contains articles on many of the points above.

Software and code

Policy information about availability of computer code

Data collection	All experimental protocols were programmed with the MATLAB-based PsychotoolBox.
Data analysis	EEG analysis was done by using MATLAB toolboxes: EEGLAB, ADJUST, CSD toolbox, FieldTrip, and N-way toolbox (details provided in Methods). MATLAB code for model computation is available in Zenodo (https://doi.org/10.5281/zenodo.7084568).

For manuscripts utilizing custom algorithms or software that are central to the research but not yet described in published literature, software must be made available to editors and reviewers. We strongly encourage code deposition in a community repository (e.g. GitHub). See the Nature Portfolio guidelines for submitting code & software for further information.

Data

Policy information about availability of data

All manuscripts must include a data availability statement. This statement should provide the following information, where applicable:

- Accession codes, unique identifiers, or web links for publicly available datasets
- A description of any restrictions on data availability
- For clinical datasets or third party data, please ensure that the statement adheres to our policy

The individual EEG data and source data underlying the figures are available from the corresponding author on reasonable request.

Human research participants

Policy information about studies involving human research participants and Sex and Gender in Research.

Reporting on sex and gender	We recruited 30 participants (self-reported sex, 15 males and 15 females) in our study, and no analysis in the separate sex groups was applied. The consent was obtained for sharing the individual data without the disclosure of personal identity.
Population characteristics	Aged between 20-30 years old (mean age: 24 +- 2.6) and has no apparent cognitive difficulties, no serious deficits in vision and hearing, and no known neurological psychological diagnosis.
Recruitment	Via advertisement post on the campus board and social media (Facebook), which would be easily access to people at young age.
Ethics oversight	The research protocols were approved by the Research Ethics Committee of the National Taiwan University Hospital.

Note that full information on the approval of the study protocol must also be provided in the manuscript.

Field-specific reporting

Please select the one below that is the best fit for your research. If you are not sure, read the appropriate sections before making your selection.

☒ Life sciences ☐ Behavioural & social sciences ☐ Ecological, evolutionary & environmental sciences

For a reference copy of the document with all sections, see nature.com/documents/nr-reporting-summary-flat.pdf

Life sciences study design

All studies must disclose on these points even when the disclosure is negative.

Sample size	The sample size in the current study (N=30) was determined based on previous EEG/MEG studies with similar design (N=29 in Bekinschtein et al., 2009; N = 10 in Wacongne et al., 2011).<br><br>Bekinschtein, T. A., Dehaene, S., Rohaut, B., Tadel, F., Cohen, L., & Naccache, L. (2009). Neural signature of the conscious processing of auditory regularities. Proceedings of the National Academy of Sciences, 106(5), 1672–1677. https://doi.org/10.1073/pnas.0809667106<br><br>Wacongne, C., Labyt, E., van Wassenhove, V., Bekinschtein, T., Naccache, L., & Dehaene, S. (2011). Evidence for a hierarchy of predictions and prediction errors in human cortex. Proceedings of the National Academy of Sciences, 108(51), 20754–20759. https://doi.org/10.1073/pnas.1117807108
Data exclusions	No data from any participant was excluded. For each participant, an average of ~2.6% of the total 2,304 trials were excluded (mean: 60.9 ± 73.9 trials) based on EEG data quality check (visual inspection).
Replication	The reproducibility of the current results were verified by rigorous statistical testing (details in Methods).
Randomization	N/A. Our study used a within-group design (i.e., single group with multiple conditions).
Blinding	N/A. Our study used a within-group design (i.e., single group with multiple conditions).

Reporting for specific materials, systems and methods

We require information from authors about some types of materials, experimental systems and methods used in many studies. Here, indicate whether each material, system or method listed is relevant to your study. If you are not sure if a list item applies to your research, read the appropriate section before selecting a response.

Materials & experimental systems

n/a	Involved in the study	
☒	☐	Antibodies
☒	☐	Eukaryotic cell lines
☒	☐	Palaeontology and archaeology
☒	☐	Animals and other organisms
☒	☐	Clinical data
☒	☐	Dual use research of concern

Methods

n/a	Involved in the study	
☒	☐	ChIP-seq
☒	☐	Flow cytometry
☒	☐	MRI-based neuroimaging
